# Epicardial Adipose Tissue CT Radiomics Improves Acute Coronary Syndrome Prediction Beyond Coronary Artery Calcium Score

**DOI:** 10.3390/diagnostics16091270

**Published:** 2026-04-23

**Authors:** Eric Po-Yu Huang, Yi-Chun Chen, Ming-Ting Wu, Jyh-Cheng Chen

**Affiliations:** 1 Department of Radiology, Kaohsiung Veterans General Hospital, No. 386, Ta-Chung 1st Road, Kaohsiung 813414, Taiwan; erich0600@gmail.com (E.P.-Y.H.); jumpjump612@gmail.com (Y.-C.C.); 2 Department of Biomedical Imaging and Radiological Sciences, National Yang Ming Chiao Tung University, Taipei 112304, Taiwan; 3 Department of Radiology, New Taipei City Hospital, No. 3, Sec. 1, New Taipei Blvd., Sanchong Dist., New Taipei City 241204, Taiwan; 4 Faculty of Medicine, School of Medicine, National Yang Ming Chiao Tung University, No. 155, Sec. 2, Linong Street, Taipei 112304, Taiwan; 5 Institute of Clinical Medicine, National Yang Ming Chiao Tung University, No. 155, Sec. 2, Linong Street, Taipei 112304, Taiwan; 6 Department of Medical Imaging and Radiological Sciences, China Medical University, Taichung 406040, Taiwan

**Keywords:** acute coronary syndrome, computed tomography, coronary artery disease, propensity score, radiomics, epicardial adipose tissue

## Abstract

**Objectives**: To determine if global epicardial adipose tissue (EAT) radiomics, derived from non-contrast coronary artery calcium (CAC) scans, improves acute coronary syndrome (ACS) prediction beyond traditional risk factors (TRFs) and Agatston score (AS) in individuals without angina. **Methods:** We retrospectively analyzed 2020 subjects without angina who underwent CAC scans from 2016 to 2019, among whom 76 patients developed acute coronary syndrome (ACS) during a follow-up period until December 2023. One-to-one propensity score matching (PSM) based on TRFs (age, sex, BMI, smoking, diabetes, hypertension, dyslipidemia) and Agatston score ranks (ASR) created 76 ACS and 76 matched non-ACS subjects. A radiomics model was built using 5-fold cross-validation on the matched cohort and tested on the entire unmatched cohort. Statistical tests included AUC comparison. **Results**: PSM effectively mitigated disparities of TRFs and ASR. The radiomics model achieved AUCs of 0.91 ± 0.01 and 0.89 ± 0.03 for the training and matched test sets, respectively, and 0.89 ± 0.03 on the unmatched cohort. The radiomics score significantly improved ACS prediction over TRF and CAC models (*p* < 0.001) in both matched and unmatched cohorts. **Conclusions**: Global EAT radiomic phenotypes from conventional CAC scan improve ACS risk stratification beyond TRFs and AS in individuals without angina. In contrast to PCAT on CCTA, our simple approach appears to be suitable for large-scale applications using preventative CAC scans.

## 1. Introduction

Acute Coronary Syndrome (ACS) is a major contributor to cardiovascular disease-related mortality worldwide, underscoring the importance of primary prevention of coronary artery disease (CAD). At present, ACS risk prediction predominantly relies on traditional risk factors (TRF), the quantification of coronary artery calcium (CAC) [1,2] using the Agatston score (AS) [3], and coronary computed tomography angiography (CCTA) [4,5,6]. While CCTA is an established non-invasive imaging modality for evaluating CAD, it requires greater resources and expertise and exposes patients to contrast administration and higher radiation than non-contrast CAC scanning. CAC scans, performed using ECG-gated non-contrast computed tomography (CT), have become a valuable tool for cardiovascular risk assessment [1,4], because they provide a surrogate marker of coronary atherosclerotic plaque burden. However, this approach may not fully capture the multifactorial nature of ACS, particularly in patients with zero calcifications [7,8]. Therefore, additional information embedded in CAC scans beyond calcification itself may provide incremental value for ACS risk prediction.

Epicardial adipose tissue (EAT) is the surrounding fat inside the pericardium. Previous studies have shown that an increase in EAT is associated with heightened inflammatory activity [9], which contributes to the development and progression of coronary atherosclerosis [10,11]. Changes in EAT attenuation are also linked to a higher risk of ACS [12,13,14]. Beyond the volume and attenuation of EAT [11,13], recent advanced CT analysis, such as radiomics, may improve the prediction of ACS.

Our research aims to develop an innovative predictive model that leverages the radiomic phenotype of EAT extracted from a small, propensity score-matched (PSM) cohort to simulate a randomized controlled trial environment for more accurate ACS risk prediction. We seek to demonstrate that even a carefully selected, small subset can yield marked improvements in risk stratification that extend to the entire cohort.

## 2. Materials and Methods

**Figure 1:**
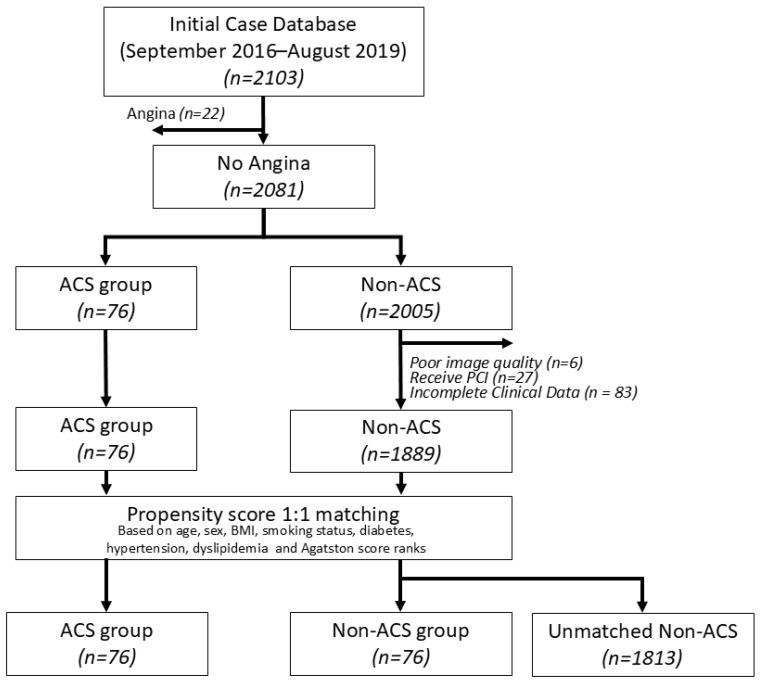
Study cohort selection and propensity score matching flowchart.

The radiological information system of our institute was queried for cardiac CT exams from September 2016 to August 2019. As illustrated in Figure 1, a total of 2103 patients were initially identified. Among these, 22 patients with angina symptoms were excluded, leaving 2081 patients without angina. After excluding 22 patients presenting with angina symptoms at baseline, 2081 asymptomatic individuals were included in the study cohort.

During a five-year follow-up period, 76 patients developed acute coronary syndrome (ACS), while 2005 did not. ACS was defined as a composite of ST-elevation myocardial infarction (STEMI), non-ST-elevation myocardial infarction (NSTEMI), and unstable angina, based on clinical diagnosis. Among ACS cases, NSTEMI accounted for 59.2%, STEMI for 25.0%, and unstable angina for 15.8%. Events were ascertained through a systematic review of electronic medical records, including emergency department visits, hospital admissions, and cardiology follow-up. Diagnostic confirmation was supported by cardiac biomarker elevation (troponin), electrocardiographic findings, and/or coronary angiography or revascularization procedures when available. Patients without documented ACS events were censored at their last known clinical follow-up.

Exclusion criteria were applied to both groups: patients with poor image quality (*n* = 6), prior percutaneous coronary intervention or coronary stent placement (*n* = 27), and incomplete clinical data (*n* = 83) were removed. After exclusions, 1889 patients remained in the non-ACS group. Notably, no ACS patients had coronary stents prior to their CAC scan. Ultimately, 76 patients who developed ACS and 1889 subjects without ACS were included for further analysis.

This flowchart depicts the selection process of the study cohort from the initial case database (September 2016–August 2019). Patients with angina were excluded, and individuals without angina were categorized into ACS and non-ACS groups based on clinical diagnosis. Propensity score matching (1:1) was performed based on variables including age, sex, BMI, smoking status, diabetes, hypertension, dyslipidemia, and Agatston score, Agatston score ranks, resulting in final groups for post-match analysis.

Baseline variables were defined as those available at the time of the index CT scan and included: age (years), CAC score, number of plaques, number of segments with plaques, number of vessels with plaques, sex, smoking status, diabetes mellitus, hypercholesterolemia, hypertension, and family history of premature cardiovascular disease. Due to the retrospective nature of the study, complete vital signs and laboratory data were not consistently available; therefore, a clinical-variable framework commonly adopted in cardiovascular imaging studies was used as the reference model [15].

PSM-based 1:1 matching resulted in 76 ACS patients matched to 76 non-ACS patients with balanced baseline characteristics. The remaining unmatched cohort of 1813 non-ACS patients was retained as the control group for a secondary unmatched cohort test.

CT acquisition was performed using a 256-detector Revolution CT scanner (GE HealthCare, Chicago, IL, USA). Scans were obtained at 120 kV with automated tube current modulation (target noise index 18), gantry rotation time of 0.28 s, and 2.5 mm collimation. Images were reconstructed at 75% of the R–R interval using a medium soft-tissue kernel, with a slice thickness of 2.5 mm and a 250 mm field of view centered on the heart.

AS [3] was calculated from non-contrast CT datasets using SmartScore 4.0 (GE HealthCare, Chicago, IL, USA). All scans were reviewed for calcified plaques by an experienced CT technologist (17 years of experience) and confirmed by a thoracic radiologist (25 years of experience). A calcified plaque was defined as an area of three connected voxels with a CT number ≥ 130 Hounsfield units (HU), applying 3D connectivity criteria. Total AS, number of plaques, number of segments with plaques, and number of vessels with plaques were recorded. Based on previous prognosis indications for AS, the ranking is as follows: Rank 1 corresponds to a score of 0; Rank 2 includes scores between 1 and 100; Rank 3 covers scores from 101 to 400; and Rank 4 is assigned to scores greater than 401 [1,2].

**Figure 2:**
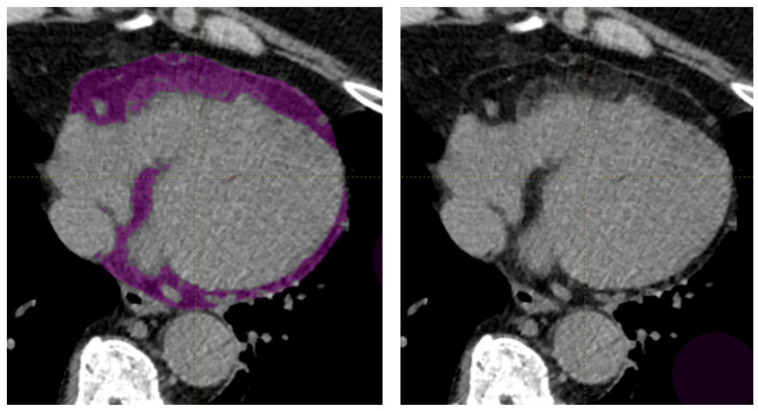
Epicardial adipose tissue (EAT) segmentation on cardiac CT. The EAT volume is highlighted in purple (left panel). The unsegmented image is shown on the right panel.

Using LIFEx software (version 6.3; Laboratory of Translational Imaging in Oncology [LITO], Institut Curie, Orsay, France) [16], the EAT was semi-automatically segmented using the software’s segmentation tools. The segmentation is based on the predefined HU range (−190 to −130 HU), accurately isolating the EAT for further analysis (Figure 2). A total of 57 radiomics features were extracted using this method, including (1) conventional and discretized HU metrics such as mean, standard deviation, coefficient of variation, quartiles, skewness, kurtosis, and excess kurtosis, (2) shape features, and (3) texture features from GLCM, GLRLM, NGLDM, and GLZLM (Table S1).

Representative cardiac CT image demonstrating segmentation of epicardial adipose tissue (EAT) in a cross-sectional view. The EAT volume is highlighted in purple (left panel). The unsegmented image is shown on the right panel.

PSM was performed to minimize the selection bias and the effects of confounders. By carefully matching patients with and without ACS based on various clinically relevant covariates, PSM helps ensure that any observed outcome differences are less likely to be driven by underlying demographic or clinical imbalances. The propensity score was calculated through the multivariable logistic regression modeling with the following covariates: age, sex, smoking status, diabetes mellitus (DM), hypercholesterolemia, hypertension, family history of cardiovascular disease, CAC score, Agatston score ranks (ASR), and number of plaques. A one-to-one matching protocol with a caliper of 0.2 was implemented to simulate a randomized controlled trial environment, ensuring close matches and minimizing residual confounding between matched subjects. The balance of covariates before and after matching was evaluated using standardized mean difference (SMD) for each covariate, and the balance improvement was visually represented using a Love plot [17].

Five predictive models were used to discriminate patients with subsequent ACS as follows:

TRFs included sex, family history of myocardial infarction, personal history of DM, hypercholesterolemia, hypertension, and smoking status. Any two features with high pairwise correlation, defined as |r| ≥ 0.9, were removed.

CAC score: We eliminated features with highly pairwise correlations, defined as |r| ≥ 0.9. Secondly, multivariate logistic regression was used to identify the significant features using the backward stepwise elimination method, and all remaining features were included in the calcification score model.

Combined CAC and traditional risk factors: Consisted of all remaining features within the TRF and CAC model.

EAT volume and attenuation: Contains total EAT volume and mean attenuation of the EAT.

Radiomics score: First, we eliminated features with highly pairwise correlations, defined as |r| ≥ 0.9.

Secondly, we applied the least absolute shrinkage and selection operator (LASSO) regression with 10-fold cross-validation to address multicollinearity and identify the most informative radiomics features, retaining those with non-zero coefficients. Finally, the selected features were entered into a multivariate logistic regression, and a radiomics score was constructed as a linear combination of these features weighted by their respective coefficients. Ten-fold cross-validation was chosen to balance model stability with computational efficiency and is widely regarded as a robust approach for preventing overfitting in feature selection.

All statistical analyses were performed using BM SPSS Statistics for Windows, version 22.0 (IBM Corp., Armonk, NY, USA) Continuous variables were presented as mean S ± SD or median (25th, 75th percentile) according to the data distribution. The chi-square test was used to compare categorical variables between two groups; either Student’s *t*-test or Mann–Whitney U test was used for the continuous variables as appropriate.

The PSM and subsequent analyses were implemented in Python (version 3.14), with libraries such as Pandas (version 3.0.1) for data manipulation, Scikit-learn (version 1.8) for logistic regression and scaling, and Matplotlib (version 3.10) for data visualization. Predictive models were developed using logistic regression. The data were standardized using a standard scaler to ensure that all features contributed equally to the model. Both test datasets were scaled using the same coefficients as the training dataset scaler to prevent data leakage.

Model performance was evaluated using stratified 5-fold cross-validation. The entire matched dataset was divided into five equal parts, with each fold maintaining the same class distribution as the overall cohort. For every iteration, 80% of the data was used for training, while the remaining 20% served as the held-out test set. The ACS cases within each 20% held-out fold were then used as a consistent case reference for performance assessment across different cohort settings. Specifically, these cases were evaluated both within the matched test set and in combination with the full unmatched cohort, allowing model performance to be examined under varying population compositions while maintaining a fixed case group. This design enables a direct comparison of model behavior between balanced (matched) and real-world (unmatched) distributions.

Model discrimination was assessed using the area under curve (AUC) of the receiver operating characteristic (ROC) curve, and different models’ AUCs were compared using DeLong’s test [18]. In the matched test set, calibration curves were plotted to evaluate the agreement between predicted probabilities and observed outcomes, and decision curve analysis (DCA) [19] was performed to assess the clinical utility of the model across a range of threshold probabilities.

## 3. Results

### 3.1. Patients’ Clinical Characteristics Before and After Matching

The patients’ clinical characteristics before and after the PSM are detailed in Table 1. The “Before Match” panel represents the full unmatched cohort (non-ACS *n* = 1889; ACS *n* = 76). In this unmatched cohort, the ACS group had markedly higher CAC scores (614 ± 1790 vs. 180 ± 607 Agatston units), greater plaque burden (number of plaques 5.28 ± 3.71 vs. 2.73 ± 6.62; segments with plaques 4.21 ± 2.66 vs. 2.17 ± 8.72; vessels with plaques 2.22 ± 1.10 vs. 1.24 ± 2.75; all *p* < 0.01), and higher prevalences of male sex (89.5% vs. 64.6%) and smoking (67.1% vs. 21.8%; both *p* < 0.01); age and diabetes were similar. After PSM, the matched cohorts (ACS *n* = 76; non-ACS *n* = 76) were well balanced across baseline variables, including CAC score (614 ± 1790 vs. 473 ± 781; *p* = 0.72), plaque burden metrics (*p* = 0.34–0.46), sex (both 89.5%; *p* = 1.00), smoking (67.1% vs. 61.8%; *p* = 0.61), diabetes (both 19.7%; *p* = 1.00), hypercholesterolemia (23.7% vs. 19.7%; *p* = 0.69), hypertension (52.6% vs. 53.9%; *p* = 1.00), and Agatston score grade distribution (*p* = 0.78).

**Figure 3:**
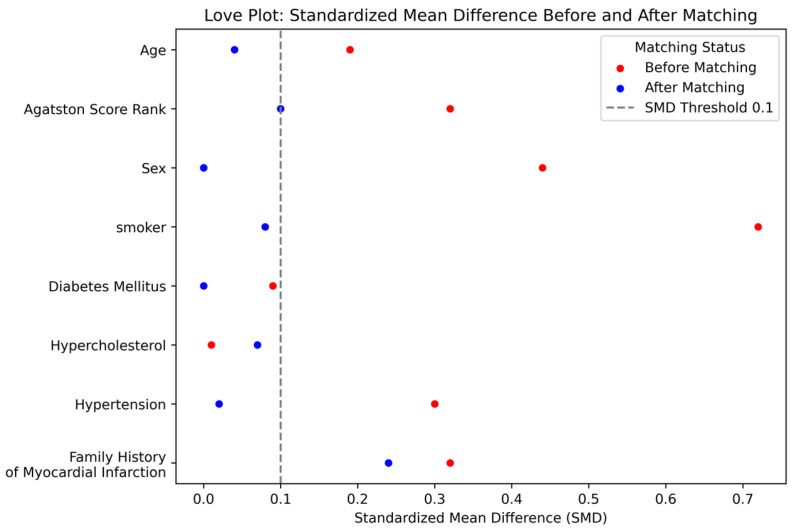
Standardized Mean Differences for Covariates Before and After Propensity Score Matching.

These differences were substantially mitigated after applying PSM, yielding no statistically significant differences between the groups in the matched dataset. Age, CAC, plaque characteristics, and demographic factors like sex and smoking status showed *p*-values well above the 0.05 threshold, indicating effective matching. The Love plot (Figure 3) illustrates the effectiveness of PSM in achieving a covariate balance between the non-ACS and ACS groups. After the matching process, SMDs were substantially reduced for all covariates, and all covariates fell within −0.1 and 0.1, which denotes the commonly accepted threshold for balance.

This Love plot visualizes the standardized mean differences (SMDs) in key covariates between non-ACS and ACS groups before and after PSM. Red dots represent the SMDs before matching, and blue dots represent the SMDs after matching. The dashed red lines at SMDs of −0.1 and 0.1 indicate the threshold for acceptable balance. The plot demonstrates effective normalization of covariate differences post-matching, highlighting the success of the matching process in controlling potential confounding factors in the comparative analysis.

### 3.2. Traditional Risk Factors and CAC Factors

No TRFs were highly correlated with each other. After the multivariable logistic regression with backward stepwise elimination was performed, age, sex, smoking status, DM, hypercholesterolemia, hypertension, and family history of myocardial infarction remained and were included in the final TRF model. For the CAC factors, the total number of plaques and the number of segments and vessels with calcification were highly correlated, and the latter two were removed. The final CAC model included the AS rank and the total number of plaques.

### 3.3. Radiomics Score

A total of 57 features were obtained from the EAT analysis and are shown in Table S1. After removing highly pairwise correlated features, 40 features remained, and the clustered correlation matrix of all radiomics variables is shown in Figure S1. The remaining 40 features were further reduced using LASSO, and ultimately, eight significant features with non-zero coefficients were used to calculate the radiomics score. The radiomics score is calculated using the following formula: Covariance: −0.0181, Discretized Histogram Excess Kurtosis: 0.0086, Volume (mL): −0.0648, GLCM Correlation: 0.0422, GLZLM HGZE: −0.0125, GLZLM SZHGE: 0.0181, GLZLM GLNU: 0.1265, GLZLM ZLNU: −0.0129.

### 3.4. ROC Curve

The model comparison was conducted based on the AUC values for matched training, matched tests, and entire cohort test sets. The TRF model achieved a mean AUC of 0.58 ± 0.03 for matched training, 0.58 ± 0.10 for the matched test, and 0.57 ± 0.14 for the entire test set, indicating relatively low predictive performance. The CAC model showed similar results, with a mean AUC of 0.58 ± 0.03 for matched training, 0.51 ± 0.08 for the matched test, and 0.54 ± 0.18 for the entire cohort test set. The TRF and CAC combined model showed slight improvement, with a mean AUC of 0.63 ± 0.03 for matched training, 0.58 ± 0.08 for the matched test, and 0.58 ± 0.11 for the entire cohort test set. The EAT volume and attenuation model showed a mean AUC of 0.56 ± 0.02 for matched training, 0.53 ± 0.08 for the matched test, and 0.63 ± 0.04 for the entire cohort test set.

**Figure 4:**
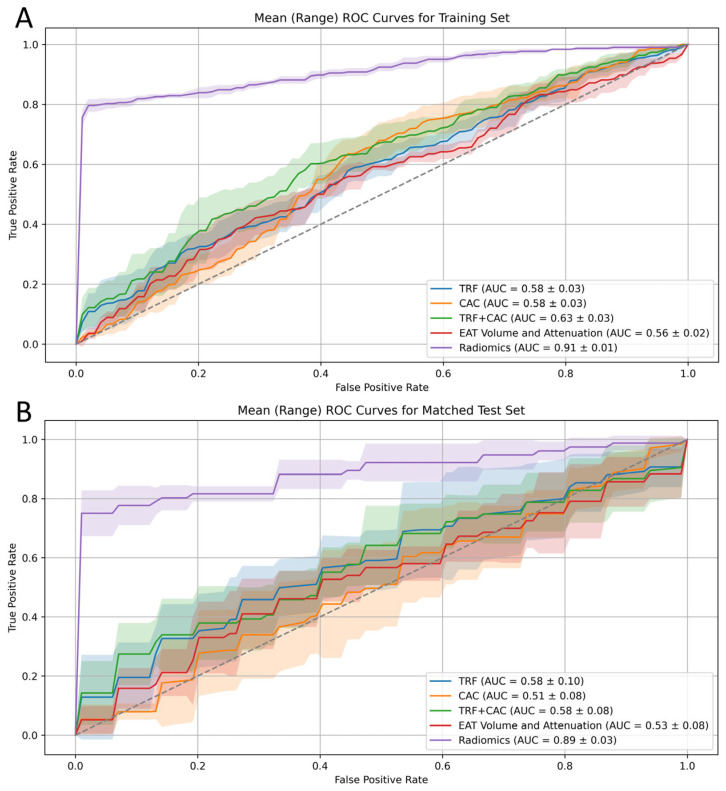
Post-Propensity Match ROC Curves for Risk Prediction Models on the Matched Training and Test Sets. Receiver operating characteristic curves compare the predictive performance of different ACS risk models in the training set (**A**) and matched test set (**B**). The radiomics model significantly outperformed all others, with an AUC of 0.91 ± 0.01 in the training set and 0.89 ± 0.03 in the matched test set (*p* < 0.01 for all comparisons). The shaded areas represent the 95% confidence interval of model performance across cross-validation folds. The dotted diagonal line indicates the line of no discrimination (AUC = 0.5).

**Figure 5:**
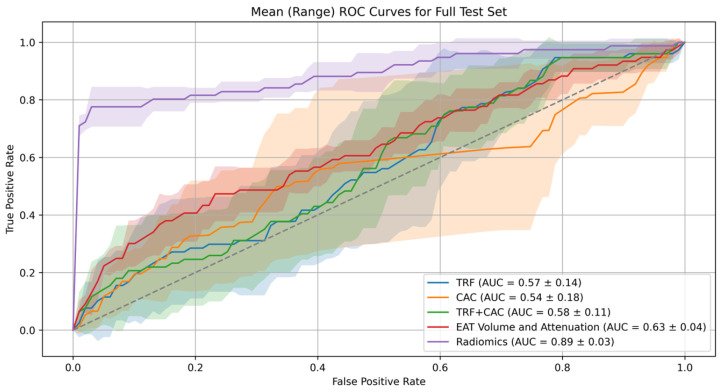
ROC Curves for Risk Prediction Models on the Test Set. The receiver operating characteristic curves for the complete unmatched test set compare the predictive performance of various risk models for ACS. The radiomics model demonstrated significantly better performance with an AUC of 0.89 ± 0.03 (*p* < 0.01 for all). The shaded areas represent the 95% confidence interval of model performance across cross-validation folds. The dotted diagonal line indicates the line of no discrimination (AUC = 0.5).

The radiomics model exhibited significantly better performance, with a mean AUC of 0.91 ± 0.01 for matched training, 0.89 ± 0.03 for the matched test, and 0.89 ± 0.03 for the complete cohort test set, substantially outperforming the other models. ROC curves for each model on the training, matched test, and complete cohort test sets are presented in Figure 4 and Figure 5. Full training and test results for each fold of the cross-validation are documented in Table S2a,b.

The mean optimal threshold across folds was 0.674 ± 0.197, yielding a sensitivity of 0.777 ± 0.066 and a specificity of 0.987 ± 0.027 (Youden index: 0.763 ± 0.067).

### 3.5. Statistical Comparison

DeLong’s test revealed that, in both the matched training and test sets, the radiomics model significantly outperformed all four other models (all *p* < 0.01). Additionally, the radiomics model demonstrated superior performance compared to all other models on the unmatched entire test cohort (*p* < 0.01). These findings suggest that the radiomics model, developed using the small-matched dataset, offers incremental benefits over CAC and TRF models, as well as the EAT model that contained only volume and mean attenuation.

### 3.6. Clinical Value of the Radiomics Score

**Figure 6:**
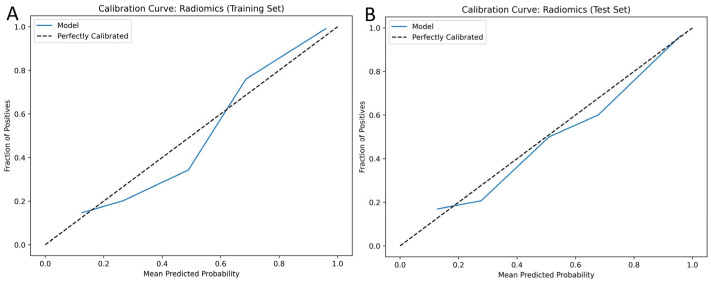
Calibration curves of the radiomics model. Calibration curves for the radiomics model illustrate the agreement between predicted probabilities and observed outcomes in the training set (**A**) and test set (**B**). The dashed line represents perfect calibration, while the solid blue line indicates model predictions. Minor under-prediction at mid-to-high predicted probabilities was observed in the test set. Hosmer–Lemeshow goodness-of-fit tests (*p* > 0.05) indicated overall acceptable calibration for both datasets.

The calibration curves (Figure 6) for radiomics and combined predictive models showed overall agreement between predicted and observed probabilities for ACS. Minor under-prediction was observed at mid-to-high probability ranges in the test set. The Hosmer–Lemeshow [20] goodness-of-fit test was non-significant (*p* > 0.05) in training and both test datasets, indicating acceptable calibration. The DCA (Figure S2) for the radiomics model showed a significant net benefit across a broad range of clinically relevant threshold probabilities, indicating that the radiomics model offers reliable predictions and offers valuable support for decision-making in ACS risk stratification.

## 4. Discussion

### 4.1. Main Findings

This study developed and validated an EAT radiomics score based on CAC scan for predicting ACS. The model built using a small, matched cohort showed incremental benefit to CAC, TRF, and combined models, as well as the EAT volume and attenuation model in predictive performance and demonstrated consistent performance across both matched and unmatched cohort settings. This design allowed assessment of model behavior under different population compositions (balanced vs. real-world distributions), supporting its potential clinical applicability.

### 4.2. Impact of Propensity Score Matching

In observational studies, differences in baseline characteristics can bias outcomes. Factors such as age, sex, and significant cardiovascular risk factors (e.g., diabetes, hypertension) may impact imaging features and the ACS risk, potentially leading to false conclusions about the predictive model efficacy. We addressed these confounders using PSM to balance key covariates between groups [17,21], simulating randomization by pairing similar patients, thereby enabling evaluation of radiomics features under controlled conditions with minimized confounding. While PSM reduces bias and enhances internal validity, it inherently limits the sample size and may not fully reflect real-world heterogeneity. The unmatched cohort was therefore used to evaluate model performance under real-world population distributions, allowing comparison between balanced (matched) and heterogeneous (unmatched) settings using a consistent case reference. Notably, the EAT volume and attenuation model showed improved performance in the unmatched cohort compared with the matched cohort, likely reflecting restoration of baseline clinical variability after matching. This suggests that conventional EAT metrics are more sensitive to underlying cardiometabolic differences, whereas radiomics features retain discriminative value under controlled conditions.

### 4.3. Cohort Discrepancies and Screening Implications

The absence of significant differences in age or prevalence of diabetes and hypercholesterolemia between the ACS and non-ACS groups initially appears counterintuitive, given that these factors are established risk markers for CAD. However, this can be attributed to the nature of our cohort, which primarily comprises individuals undergoing self-paid health screening. Such screenings are often sought by individuals with a heightened perception of cardiovascular risk or advancing age, leading to a cohort with inherently elevated risk profiles despite being categorized as asymptomatic. This category of patients aligns with the 2023 Taiwan Society of Cardiology guideline, recommending CAC scans for asymptomatic patients at low to moderate risk [4].

### 4.4. Rationale for Using CAC Scans over CCTA

While CCTA scans are widely used for EAT radiomic analyses [22,23,24], CAC scans were chosen for this study for several compelling reasons. First, according to the consensus of primary preventative cardiology, a CAC scan is the recommended examination for risk stratification. It is more widely available in healthcare check-up programs. Unlike CCTA, CAC scans do not require contrast media, reducing variability associated with contrast timing, dose, or beam-hardening artifacts caused by high-density iodine, which may affect the consistency of radiomic feature extraction [25,26].

### 4.5. Comparison of EAT and PCAT as Biomarkers

EAT, the adipose tissue surrounding the myocardium and coronary arteries without intervening barriers, is a marker of global inflammation and CAD [12,27]. PCAT, generally defined as the local adipose tissue surrounding the proximal 4 cm of major coronary arteries, is a subset of EAT and has recently been recognized as a relevant biochemical predictor of CAD and ACS. However, PCAT evaluation requires sophisticated software that is not widely available, and its segmentation is labor-intensive and dependent on user expertise [28]. In contrast, EAT segmentation is more straightforward, reproducible, and adaptable across diverse imaging protocols, making it a more practical choice for broader applications [13,29]. This distinction highlights EAT radiomics as a scalable and clinically accessible alternative that complements PCAT-based approaches.

### 4.6. Radiomics Features in ACS Prediction

Our findings align with previous research demonstrating that EAT volume and fat attenuation index are associated with CAD and major adverse cardiovascular events, even among young adults [10,11,13]. However, this study is among the first to underscore the added predictive value of radiomics features beyond simple volume measurements in predicting ACS using standard CAC scans. Radiomics features associated with ACS risk in our score cluster into complementary domains: (a) intensity dispersion/joint variability (covariance), which may reflect voxel-level heterogeneity of EAT composition; (b) distribution shape (discretized histogram excess kurtosis), indexing heavy-tailed attenuation distributions that could arise from sparse higher-attenuation foci; (c) texture/structural organization (GLCM correlation and GLZLM metrics—HGZE, SZHGE, GLNU, ZLNU), characterizing how gray levels cluster into zones and how uniform those zones are; and (d) size (EAT volume). In our fitted model, positive coefficients for GLZLM GLNU, GLCM correlation, GLZLM SZHGE, and excess kurtosis indicate that greater heterogeneity and small high-attenuation zones are associated with higher predicted ACS risk, whereas negative coefficients for GLZLM HGZE, GLZLM ZLNU, covariance, and volume may indicate a lower conditional contribution once correlated features are considered. For example, EAT volume showed a positive univariate association with ACS (Table S3) but a negative multivariable coefficient in the score. The negative multivariable coefficient for EAT volume, despite its positive univariate association, may reflect a conditional sign reversal in the setting of correlated radiomics features and penalized feature selection rather than a true inverse biological effect of EAT volume [30]. In this context, texture and intensity metrics may capture aspects of EAT heterogeneity beyond overall size. However, the exact biological and clinical interpretation of individual radiomic features remains challenging [31]. Our findings support the premise that EAT radiomic phenotypes may add prognostic information beyond size alone, but the contribution of individual features should be interpreted cautiously and confirmed in larger, harmonized multicenter cohorts.

### 4.7. Comparison with Recent Studies

Our results complement and extend emerging evidence that CT-derived adipose phenotypes carry prognostic information beyond fat quantity alone. Prior work using non-contrast CAC scans has shown that EAT radiomics can predict downstream ischemia and CAD burden, supporting the feasibility of extracting informative fat texture on routine scans [32]. In parallel, studies leveraging CCTA have demonstrated that pericoronary adipose tissue (PCAT) metrics—including fat attenuation index and PCAT radiomics—associate with inflammation, vulnerable plaque characteristics, and adverse outcomes; however, these often require contrast, vessel-specific segmentation, or specialized workflows [24]. More recently, adipose “fat-omics” approaches and multicenter PCAT-radiomics cohorts have reported incremental predictive value for MACE/ACS, underscoring the clinical potential of adipose phenotyping. Compared with these studies, our work focuses on standard non-contrast CAC imaging, using ACS as the endpoint with PSM and an unmatched validation cohort and proposes that useful EAT radiomics features can be extracted without contrast or coronary tracking.

### 4.8. Limitations

This study has several limitations. First, the retrospective, single-center case–control design may introduce selection bias and limit generalizability. This retrospective design limited the availability of continuous vitals/laboratories at the index CT; we therefore used a prespecified Clinical-only benchmark and quantified the incremental value of EAT radiomics over this benchmark. Furthermore, while the sample size is modest, it was adequate for the prespecified analyses and provides a foundation for external validation in independent cohorts. Prospective multicenter studies with standardized data capture are needed to incorporate continuous variables and validated risk scores for fairer comparison. Second, although the radiomics model achieved very high AUCs in the training cohort, raising the possibility of overfitting, we mitigated this risk through feature reduction (correlation filtering and LASSO) and stratified cross-validation within the matched cohort. However, because the unmatched cohort shares ACS cases with the matched dataset, it does not constitute independent external validation. Nevertheless, independent external validation in separate cohorts will be essential to confirm generalizability. Third, while radiomics features have shown strong reproducibility in our analysis, variability in image acquisition, reconstruction, and analysis protocols reported in previous studies may impact feature consistency. Fourth, we did not account for the precise time interval between CAC imaging and ACS diagnosis, which could provide deeper insights into the temporal relationship between EAT features and ACS risk. This limitation restricts the interpretation of the model as a time-dependent risk predictor, and future studies incorporating time-to-event analyses are warranted. Lastly, although segmentation was performed using standardized HU thresholds with expert supervision, formal interobserver and intraobserver reproducibility were not assessed and should be evaluated in future studies.

## 5. Conclusions

The radiomics approach of evaluating EAT on standard non-contrast CAC scan offers a simple, non-invasive, reproducible, and scalable method for assessing ACS risk, demonstrating incremental predictive performance to the TRF, CAC and combined models. Our results indicate that radiomics features of EAT alone carry significant predictive value and improve the clinical utility of standard CAC scans. Integrating these features with clinical and lifestyle factors could enable a more comprehensive cardiovascular risk assessment. These advancements could support personalized medicine initiatives and enhance population health management strategies.

## Figures and Tables

**Table 1:** Baseline Characteristics of ACS and Non-ACS Cohorts Before and After Propensity Score Matching.

Characteristics	Before Match			After Match		
	Non-ACS Cohort (*n* = 1889)	ACS Cohort (*n* = 76)	*p* Value	Non-ACS Cohort (*n* = 76)	ACS Cohort (*n* = 76)	*p* Value
Age (years)	57.47 ± 9.05	59.57 ± 13.11	0.23	59.99 ± 9.42	59.57 ± 13.11	0.77
CAC score	180 ± 607	614 ± 1790	<0.01 *	473 ± 781	614 ± 1790	0.72
Number of plaque (*n*)	2.73 ± 6.62	5.28 ± 3.71	<0.01 *	4.86 ± 4.26	5.28 ± 3.71	0.34
Segments with plaques (*n*)	2.17 ± 8.72	4.211 ± 2.66	<0.01 *	3.47 ± 2.69	4.21 ± 2.66	0.09
Vessels with plaques (*n*)	1.24 ± 2.75	2.22 ± 1.10	<0.01 *	2.08 ± 1.27	2.22 ± 1.10	0.46
Sex (male, %)	1221 (64.6%)	68 (89.5%)	<0.01 *	68 (89.5%)	68 (89.5%)	1
Smoker (*n*, %)	412 (21.8%)	51 (67.1%)	<0.01 *	47 (61.8%)	51 (67.1%)	0.61
DM (*n*, %)	473 (25.0%)	15 (19.7%)	0.36	15 (19.7%)	15 (19.7%)	1
Hypercholesterolemia (*n*, %)	438 (23.2%)	18 (23.7%)	1	15 (19.7%)	18 (23.7%)	0.69
Hypertension (*n*, %)	606 (32.1%)	40 (52.6%)	<0.01 *	41 (53.9%)	40 (52.6%)	1
Family History (*n*, %)	372 (19.7%)	4 (5.3%)	0.03 *	0.0 (0.0%)	4 (5.3%)	0.13
Agatston Score Grade			<0.01 *			0.78
0	931 (49.3%)	8 (10.5%)		11 (14.5%)	8 (10.5%)	
1–100	483 (25.6%)	26 (34.2%)		22 (28.9%)	26 (34.2%)	
100–300	252 (13.3%)	16 (21.1%)		14 (18.4%)	16 (21.1%)	
300+	223 (11.8%)	26 (34.2%)		29 (38.2%)	26 (34.2%)	

ACS: Acute coronary syndrome, CAC: Coronary artery calcium, DM: Diabetes Mellitus. * *p* < 0.05 was considered statistically significant.

## Data Availability

The original contributions presented in this study are included in the article/Supplementary Material. Further inquiries can be directed to the corresponding authors.

## Supplementary Materials

The following supporting information can be downloaded at: https://www.mdpi.com/article/10.3390/diagnostics16091270/s1, Table S1. Radiomic Features Used in Machine Learning Classifiers; Figure S1: Clustered Correlation Matrix of All Radiomics Variables; Table S2. (a): Full training and Test Results for Each Fold of the Cross-validation; (b): Full Cohort Test Results for Each Fold of the Cross-validation; Figure S2: Decision curve analysis (DCA) of the radiomics model for ACS prediction; Table S3: Non-parametric Univariable Test for Radiomic Features.

## Abbreviations

The following abbreviations are used in this manuscript:

ACS	Acute Coronary Syndrome
CAD	Coronary Artery Disease
TRF	Traditional Risk Factors
CAC	Coronary Artery Calcium
AS	Agatston Score
CCTA	Coronary Computed Tomography Angiography
ECG	Electrocardiogram
CT	Computed Tomography
EAT	Epicardial Adipose Tissue
PSM	Propensity Score-Matched
DM	Diabetes Mellitus
ASR	Agatston Score Ranks
SMD	Standardized Mean Difference
LASSO	Least Absolute Shrinkage and Selection Operator
SD	Standard Deviation
AUC	Area Under Curve
ROC	Receiver Operating Characteristic
DCA	Decision Curve Analysis
HU	Hounsfield unit
PCAT	Perivascular Adipose Tissue
PCI	Percutaneous Coronary Intervention
