# Supplementary material for: Epicardial Adipose Tissue CT Radiomics Improves Acute Coronary Syndrome Prediction Beyond Coronary Artery Calcium Score
Source: Diagnostics (Basel). 2026 Apr 23;16(9):1270. doi: 10.3390/diagnostics16091270 (PMC13162853; doi:10.3390/diagnostics16091270)
Supplement: Supplementary file 1 [file diagnostics-16-01270-s001.zip › Diagnostics Supplementary.pdf]

Table S1. Radiomic Features Used in Machine Learning Classifiers

Feature Category	Features
Shape (n = 1)	SHAPE Volume (mL)
First-order statistics (n = 24)	Conventional HU Mean, Conventional HU Standard Deviation, Conventional Covariance, Conventional HU First Quartile, Conventional HU Median, Conventional HU Third Quartile, Conventional HU Skewness, Conventional HU Kurtosis, Conventional HU Excess Kurtosis, Discretized HU Mean, Discretized HU Standard Deviation, Discretized Covariance, Discretized HU First Quartile, Discretized HU Median, Discretized HU Third Quartile, Discretized HU Skewness, Discretized HU Kurtosis, Discretized HU Excess Kurtosis, Discretized Histogram Skewness, Discretized Histogram Kurtosis, Discretized Histogram Excess Kurtosis, Discretized Histogram Entropy log10, Discretized Histogram Entropy log2, Discretized Histogram Energy Uniformity.
GLCM: Gray Level Co-occurrence Matrix (n = 7)	Homogeneity Inverse Difference, Energy Angular Second Moment, Contrast Variance, Correlation, Entropy log10, Entropy log2 Joint Entropy, Dissimilarity
GLRLM: Gray Level Run Length Matrix (n = 11)	**SRE** - Short Run Emphasis, **LRE** - Long Run Emphasis, **LGRE** - Low Gray-Level Run Emphasis, **HGRE** - High Gray-Level Run Emphasis, **SRLGE** - Short Run Low Gray-Level Emphasis, **SRHGE** - Short Run High Gray-Level Emphasis, **LRLGE** - Long Run Low Gray-Level Emphasis, **LRHGE** - Long Run High Gray-Level Emphasis, **GLNU** - Gray-Level Non-Uniformity, **RLNU** - Run Length Non-Uniformity, **RP** - Run Percentage,
NGLDM: Neighborhood Gray-Level Different Matrix (n=3)	Coarseness, Contrast, Busyness
GLZLM: Gray Level Size Zone Matrix (n = 11)	**SZE** - Small Zone Emphasis, **LZE** - Large Zone Emphasis, **LGZE** - Low Gray-Level Zone Emphasis, **HGZE** - High Gray-Level Zone Emphasis, **SZLGE** - Small Zone Low Gray-Level Emphasis, **SZHGE** - Small Zone High Gray-Level Emphasis, **LZLGE** - Large Zone Low Gray-Level Emphasis, **LZHGE** - Large Zone High Gray-Level Emphasis, **GLNU** - Gray-Level Non-Uniformity, **ZLNU** - Zone Length Non-Uniformity, **ZP** - Zone Percentage,

HU: Hounsfield units

Figure S1: Clustered Correlation Matrix of All Radiomics Variables

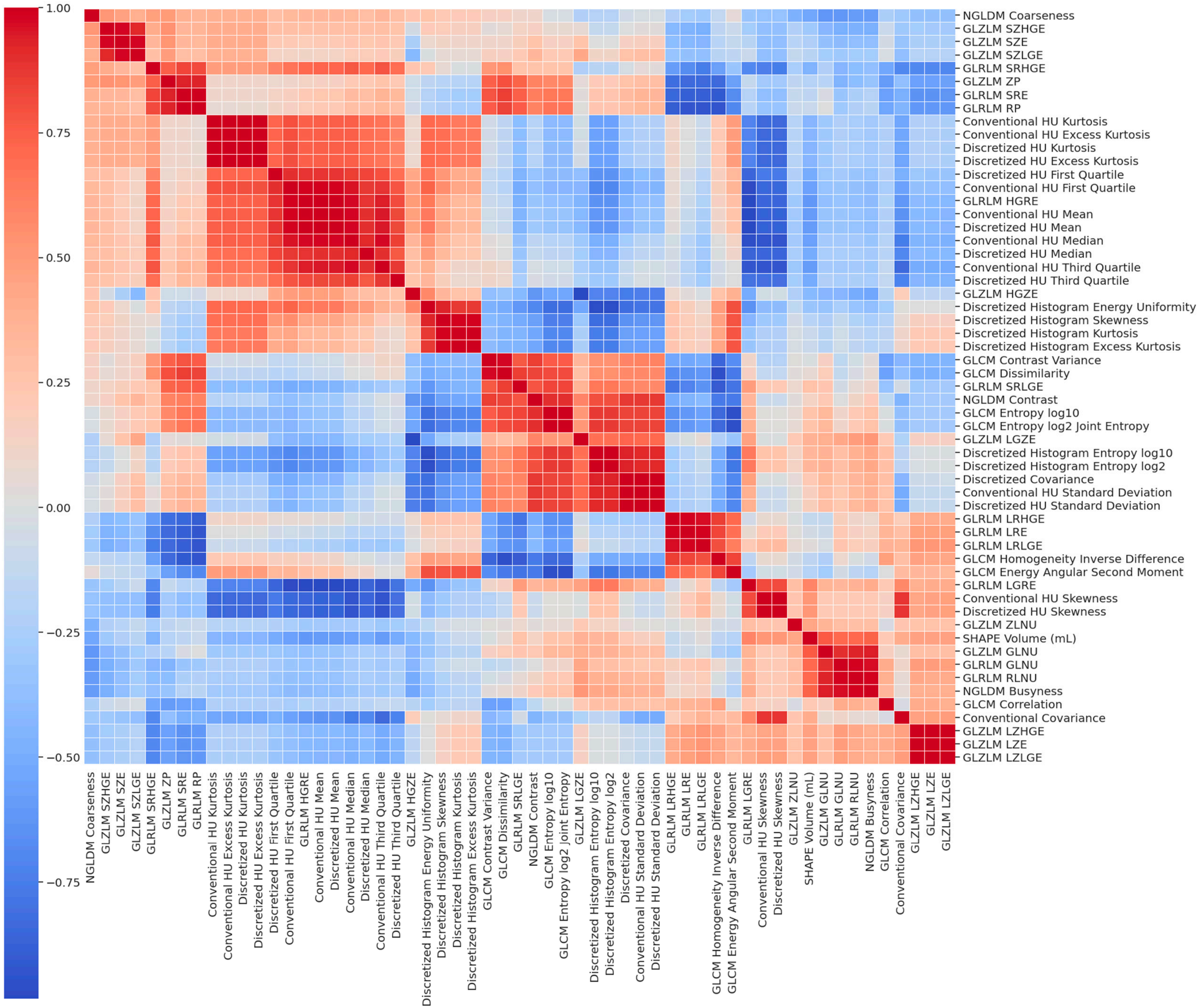

Table S2a: Full training and Test Results for Each Fold of the Cross-validation

	Training					Matched Test				
	TRF	CAC	TRF+CAC	EAT HU + Vol	Radiomics	TRF	CAC	TRF+CAC	EAT HU + Vol	Radiomics
Fold 1	0.635	0.554	0.653	0.579	0.915	0.263	0.246	0.425	0.450	0.246
Fold 2	0.583	0.561	0.608	0.537	0.922	0.448	0.45	0.45	0.662	0.4
Fold 3	0.565	0.507	0.567	0.553	0.91	0.309	0.413	0.611	0.551	0.462
Fold 4	0.573	0.577	0.604	0.557	0.909	0.520	0.422	0.498	0.462	0.449
Fold 5	0.559	0.471	0.571	0.555	0.905	0.238	0.549	0.327	0.524	0.447
Mean	0.580	0.530	0.60	0.56	0.91	0.42	0.46	0.40	0.53	0.89

TRF: Traditional risk factors; CAC: Coronary artery calcification; EAT HU + Vol: Epicardial adipose tissue attenuation and volume

Table S2b: Full Cohort Test Results for Each Fold of the Cross-validation

	Unmatched Cohort Test				
	TRF	CAC	TRF+CAC	EAT HU + Vol	Radiomics
Fold 1	0.186	0.78	0.26	0.631	0.884
Fold 2	0.475	0.745	0.583	0.689	0.841
Fold 3	0.396	0.669	0.491	0.597	0.909
Fold 4	0.496	0.67	0.536	0.579	0.924
Fold 5	0.593	0.253	0.492	0.658	0.907
Mean	0.43	0.62	0.47	0.63	0.89

TRF: Traditional risk factors; CAC: Coronary artery calcification; EAT HU + Vol: Epicardial adipose tissue attenuation and volume

Figure S2: Decision curve analysis (DCA) of the radiomics model for ACS prediction.

Figure Captions: The decision curve illustrates the net benefit of the radiomics model (green) compared to the treat-all strategy (red) and treat-none strategy (blue) across a range of threshold probabilities. The x-axis represents the threshold probability for intervention, while the y-axis denotes net benefit. The radiomics model demonstrates superior net benefit across clinically relevant thresholds, supporting its potential clinical utility in ACS risk stratification.

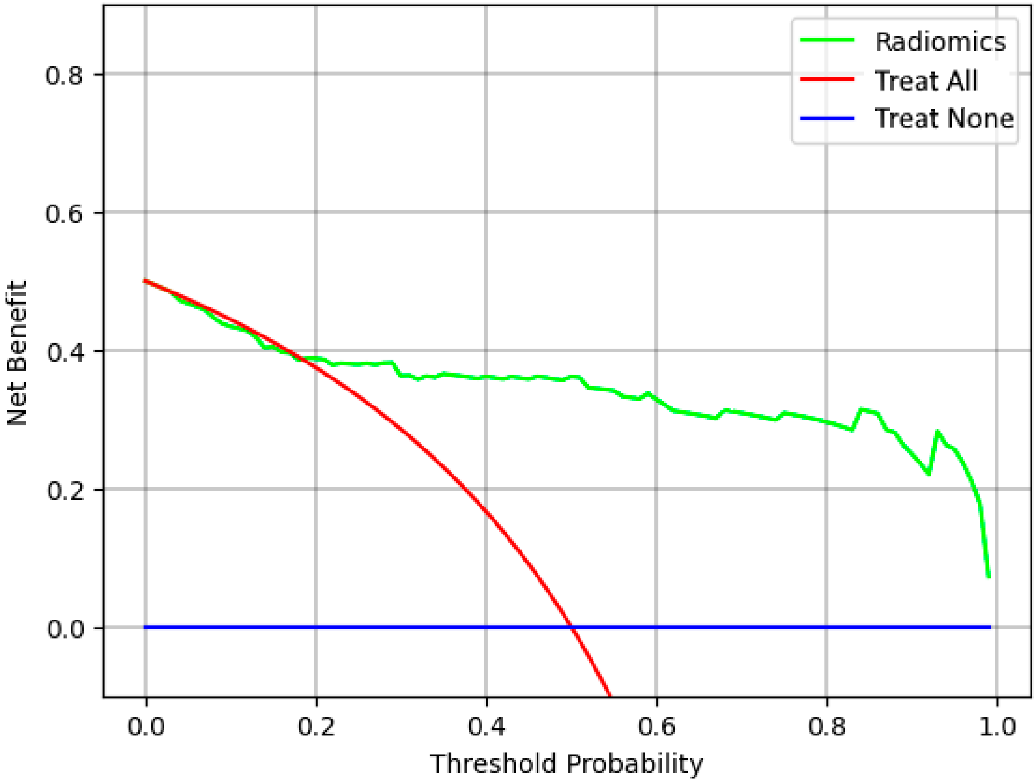

Table S3: Non-parametric Univariable Test for Radiomic Features

Radiomic variable	Non-ACS	ACS	p-value
Conventional HU Mean	-80.67	-80.15	0.497
Conventional HU Standard Deviation	28.9	31.8	<0.001*
Conventional Covariance	-0.36	-0.4	<0.001*
Conventional HU First Quartile	-101	-102	0.428
Conventional HU Median	-81	-77	0.001*
Conventional HU Third Quartile	-58	-54	<0.001*
Conventional HU Skewness	-0.29	-0.52	<0.001*
Conventional HU Kurtosis	2.79	2.83	0.227
Conventional HU Excess Kurtosis	-0.21	-0.17	0.227
Discretized HU Mean	92.48	92.53	0.513
Discretized HU Standard Deviation	2.9	3.19	<0.001*
Discretized Covariance	0.03	0.03	<0.001*
Discretized HU First Quartile	90	90	0.808
Discretized HU Median	92	93	0.002
Discretized HU Third Quartile	95	95	<0.001*
Discretized HU Skewness	-0.29	-0.51	<0.001*
Discretized HU Kurtosis	2.78	2.82	0.221
Discretized HU Excess Kurtosis	-0.22	-0.18	0.221
Discretized Histogram Skewness	6.6	6.42	<0.001*
Discretized Histogram Kurtosis	46.16	43.71	<0.001*
Discretized Histogram Excess Kurtosis	43.16	40.71	<0.001*
Discretized Histogram Entropy log10	1.05	1.08	<0.001*
Discretized Histogram Entropy log2	3.49	3.59	<0.001*
Discretized Histogram Energy Uniformity	0.1	0.09	<0.001*
SHAPE Volume mL	120.8	138.92	0.001*
GLCM Homogeneity Inverse Difference	0.46	0.46	0.159
GLCM Energy Angular Second Moment	0.01	0.01	<0.001*
GLCM Contrast Variance	8.15	8	0.186
GLCM Correlation	0.45	0.57	<0.001*
GLCM Entropy log10	1.98	2.04	<0.001*
GLCM Entropy log2 Joint Entropy	6.58	6.77	<0.001*
GLCM Dissimilarity	2.1	2.1	0.58
GLRLM SRE	0.9	0.9	0.517
GLRLM LRE	1.63	1.58	0.001*
GLRLM LGRE	0	0	0.588
GLRLM HGRE	8572.44	8574.04	0.853
GLRLM SRLGE	0	0	0.341
GLRLM SRHGE	7689.96	7708.11	0.466

GLRLM LRLGE	0	0	<0.001*
GLRLM LRHGE	13879.25	13545.96	0.006*
GLRLM GLNU	18883.65	50097.52	<0.001*
GLRLM RLNU	150854.99	429086.62	<0.001*
GLRLM RP	0.86	0.86	0.367
NGLDM Coarseness	0	0.00E+00	<0.001*
NGLDM Contrast	0.09	0.1	<0.001*
NGLDM Busyness	20.12	54.64	<0.001*
GLZLM SZE	0.57	0.57	0.162
GLZLM LZE	8225.3	16504.46	<0.001*
GLZLM LGZE	0	0	<0.001*
GLZLM HGZE	8617.61	8512.36	<0.001*
GLZLM SZLGE	0	0.00E+00	<0.001*
GLZLM SZHGE	4924.11	4858.5	0.326
GLZLM LZLGE	0.99	1.88	<0.001*
GLZLM LZHGE	68500000	147430510.8	<0.001*
GLZLM GLNU	2965.86	6136.64	<0.001*
GLZLM ZLNU	10480.57	11206	0.866
GLZLM ZP	0.16	0.14	<0.001*
